# Understanding relevance of health research: considerations in the context of research impact assessment

**DOI:** 10.1186/s12961-017-0188-6

**Published:** 2017-04-17

**Authors:** Mark J. Dobrow, Fiona A. Miller, Cy Frank, Adalsteinn D. Brown

**Affiliations:** 1 Institute of Health Policy, Management and Evaluation, Dalla Lana School of Public Health, University of Toronto, 155 College Street, 4th Floor, Toronto, ON M5T 3M6 Canada; 2 Alberta Innovates - Health Solutions, Edmonton, Alberta Canada

**Keywords:** Health research systems, Research relevance, Research impact, Ontario, Canada

## Abstract

**Background:**

With massive investment in health-related research, above and beyond investments in the management and delivery of healthcare and public health services, there has been increasing focus on the impact of health research to explore and explain the consequences of these investments and inform strategic planning. Relevance is reflected by increased attention to the usability and impact of health research, with research funders increasingly engaging in relevance assessment as an input to decision processes. Yet, it is unclear whether relevance is a synonym for or predictor of impact, a necessary condition or stage in achieving it, or a distinct aim of the research enterprise. The main aim of this paper is to improve our understanding of research relevance, with specific objectives to (1) unpack research relevance from both theoretical and practical perspectives, and (2) outline key considerations for its assessment.

**Approach:**

Our approach involved the scholarly strategy of review and reflection. We prepared a draft paper based on an exploratory review of literature from various fields, and gained from detailed and insightful analysis and critique at a roundtable discussion with a group of key health research stakeholders. We also solicited review and feedback from a small sample of expert reviewers.

**Conclusions:**

Research relevance seems increasingly important in justifying research investments and guiding strategic research planning. However, consideration of relevance has been largely tacit in the health research community, often depending on unexplained interpretations of value, fit and potential for impact. While research relevance seems a necessary condition for impact – a process or component of efforts to make rigorous research usable – ultimately, relevance stands apart from research impact. Careful and explicit consideration of research relevance is vital to gauge the overall value and impact of a wide range of individual and collective research efforts and investments. To improve understanding, this paper outlines four key considerations, including how research relevance assessments (1) orientate to, capture and compare research versus non-research sources, (2) consider both instrumental versus non-instrumental uses of research, (3) accommodate dynamic temporal-shifting perspectives on research, and (4) align with an intersubjective understanding of relevance.

## Background

Various levels of government in Canada collectively invest multiple billions of dollars in health-related research per annum, above and beyond investments in the management and delivery of healthcare and public health services. In recognition of this sizeable collective commitment, much work has focused on the impact of health research to explore and explain the consequences of these investments and inform strategic planning. Relevance is tacit in the increased attention to the usability and impact of health research. Additionally, research funders increasingly engage in relevance assessment as an input to decision processes; yet, it is unclear whether relevance is a synonym for or predictor of impact, a necessary condition or stage in achieving it, or a distinct aim of the research enterprise. Therefore, the main aim of this paper is to improve our understanding of research relevance as it relates to research quality and research impact, with specific objectives to (1) unpack research relevance from both theoretical and practical perspectives, and (2) outline key considerations for the assessment of research relevance.

Globally, there has been increasing critical assessment of the value of health research investments [1–3], with growing interest in research impact assessment (RIA) in the health sector [4–6]. RIA focuses on understanding how research activity can directly and indirectly advance knowledge, influence decision-making, and effect health and socio-economic outcomes, with a small but growing body of work seeking to develop better measures to evaluate (and ideally attribute) the returns on health research investments [6]. The Canadian Academy of Health Sciences (CAHS) released a comprehensive report on the subject in 2009 that presented a call for action, with a number of recommendations including establishing collaborative efforts among Canadian research funders to advance frameworks and sets of indicators and metrics for health research impact [4]. The CAHS impact framework [4], which drew on the Buxton and Hanney [7] ‘payback model’, among others, has provided a thoughtful starting point for considering the impact of health research in Canada. Subsequent work by Alberta Innovates – Health Solutions (AIHS) on a Research to Impact Framework (described in Graham et al. [8]) provides further insights on operationalising RIA frameworks for health research in Canada.

These initiatives are part of a broadly discussed shift in approaches to knowledge production, from an emphasis on investigator-initiated, curiosity driven work judged and guided by scientists, to expanded approaches to knowledge production, drawing on a wider set of actors and approaches, and emphasising relevance and usability. This shift from science produced by and for scientists to knowledge production that is “*socially distributed, application-oriented, trans-disciplinary, and subject to multiple accountabilities*” [9] has been characterised as a shift from ‘mode 1’ to ‘mode 2’ knowledge regimes. In the language of mode 2, interest in research ‘impact’ expresses a concern for application or consequence, and – in the economic language of return on investment – a concern that the yield is at least equal to the investment in the research itself. Extending this reasoning, interest in research ‘relevance’ may reflect a concern for accountability – linking research to the actor(s) for whom the research is performed and who will, ideally, put it to use.

In Canada, interest in research impact and relevance appears to have been felt most forcefully in the context of health services and policy research, which has long been encouraged to orient to the needs of policymakers, health system planners and related decision makers. More recently, there has been increased attention to ensuring that all forms of health research are ‘patient oriented’ – that is, that the research is prioritised, conducted and applied in ways that are accountable to this important end user. This call has been picked up on several fronts, including by the Canadian Institutes of Health Research (CIHR), which released its Strategy for Patient-Oriented Research (SPOR) in 2011. The SPOR vision *“…is to demonstrably improve health outcomes and enhance patients’ health care experience through integration of evidence at all levels in the health care system*” [10]. In some respects, it represents a fundamental re-orientation for the primary funder of health research in Canada.

Though relevance is tacit in attention to research impact and the wider concern with mode 2 knowledge production, explicit attention to the meaning or measurement of research relevance is limited. The CAHS and AIHS frameworks, for example, acknowledge ‘relevance’ of health research but do not clearly define the term nor describe approaches for assessing it [4, 8]. Rather, these frameworks emphasise the role of broad stakeholder engagement approaches and feedback mechanisms as methods for addressing relevance. For example, the AIHS framework notes the challenge of, and need to, move “*…beyond the collection of traditional scientific indicators* […] *to include measures of greater interest to the broader stakeholder community…*” [8] without stating explicitly how “*greater interest*” or related concepts such as relevance should be judged. As currently constructed, these RIA frameworks provide important advances in how we think about the impact of health research, but they were not intended to provide guidance specifically to the assessment of the relevance of health research.

Despite this lack of specific guidance on research relevance from a scholarly or measurement perspective, attention to it as a practical component of health research funding and organisation is evolving. There is, for example, growing use of ‘relevance assessment’ by research funders. The Canadian Health Services Research Foundation, in particular, was an innovator in incorporating relevance review into its applied research funding programmes, including promoting partnerships and knowledge translation (KT) with health system stakeholders [11]. Current applications for funding from the Institute of Gender and Health at CIHR go through ‘relevance review’ (http://www.cihr-irsc.gc.ca/e/45212.html). Similarly, applications for Ontario’s Health System Research Fund are judged based on ‘internal review of relevance and impact’ (http://www.health.gov.on.ca/en/pro/ministry/research/cihr.aspx). However, given the lack of conceptual clarity on research relevance, and in particular, how relevance assessment aligns with and differs from impact assessment, there is a critical gap in our understanding that has implications for both its contemporary and ongoing application and our ability to make sound research investment decisions.

### Approach

This work was commissioned by the Ontario SPOR SUPPORT (Support for People and Patient-Oriented Research and Trials) Unit (OSSU) – one of several units established at provincial and regional levels across Canada to work with CIHR in pursuing the SPOR. Like other research organisations, OSSU saw the need to consider the relevance of the research it supported, and it established both scientific and relevance advisory committees as part of its original governance structure [12], tasking the latter to “*…develop a measure, or small set of strategic measures, that serves to inspire the Ontario research, implementation, provider and patient communities to come together to make a difference for patients*” [12]. In the spirit of research and scholarship, OSSU then asked what exactly this commitment to research ‘relevance’ entailed.

Our approach to answering this question involved the scholarly strategy of review and reflection. As with the early investigations into research impact assessment, we were surprised to find so little reflexive attention to the topic within the health research community [13]. We prepared a draft paper based on an exploratory review of literature from various fields, and gained from detailed and insightful analysis and critique at a roundtable discussion with a small group of key health research stakeholders. We also solicited review and feedback from a small sample of expert reviewers.

The structure of our paper is as follows. First, to ‘unpack’ the concept of relevance, we review theoretical literature and then consider practical work both from within and outside the health sector, to ask what has been argued and concluded about the nature of relevance and its appropriate assessment. Next, we outline a series of forward-looking considerations for assessing research relevance and conclude with reflections on how research relevance assessment fits with evolving interest in RIA.

### Unpacking relevance

#### Theoretical perspectives

Before considering the relevance of health research, we need to step back and consider what we mean by the term ‘relevance’. A range of descriptors is often used to define relevance, including ‘pertinent to…’, ‘bearing upon…’, ‘connected with…’, or ‘appropriate to…’, ‘…the matter at hand’, as well as ‘germane’, ‘apropos’, ‘material’, ‘applicable’ and ‘satisfactory’. A large body of dedicated theoretical work on relevance, drawn from many fields and perspectives, such as computer science, information science, statistics/probability theory, artificial intelligence, cognitive science, epistemology, linguistics and jurisprudence [14], reflects its importance but also the challenge for establishing a common understanding of the term [14, 15]. For example, Gärdenfors [16], in his discussion on the logic of relevance, noted that “*…relevance ought to be a central concept in the philosophy of science…*” given the position that “*…it is only relevant information that is of any importance…*” (p. 351). However, from a ‘research’ relevance perspective, the theoretical work on relevance has been linked to ‘information’, ‘evidence’, ‘reasoning’, ‘argument’ and ‘decision’ [15–18], each presenting variable framing that impedes practical definition or consistent comprehension of the term. Floridi [14] recently suggested that existing theories are “*…utterly useless when it comes to establish the actual relevance of some specific piece of information*” (p. 69), and goes on to advance a ‘subjectivist’ interpretation, with relevance judged by the questioner. While a subjectivist approach to relevance is intuitively appealing, its contribution to the assessment of research relevance presents particular challenges that we will discuss later in the paper.

Another approach to unpacking relevance is to consider the theoretical model behind the broad-based research strategies that have governed research investments and policies in high-income countries since the end of the Second World War. For the better part of the 20th century, a linear model was the dominant conceptual framework, whereby basic research was viewed as a necessary input for applied research, which then led to development and production [19, 20]. In the late 1990s, an alternate thesis was introduced when Stokes proposed a new model for broad-based research strategy – known as Pasteur’s Quadrant – that highlighted the conceptual relationship between the ‘quest to understand’ and ‘practical needs’ [21]. While some research is clearly focused on advances in basic research (e.g. Niels Bohr’s foundational research on atomic structure and quantum theory), and some research is clearly focused on applied problems (e.g. Thomas Edison’s practical inventions), Stokes emphasised the potential for use-inspired basic research (e.g. Louis Pasteur’s foundational research on microbiology that addressed contemporaneous population health challenges). Pasteur’s Quadrant invokes consideration of ‘relevance’ with some commentators framing the two-by-two relationship as the relevance for advancement of basic knowledge and the relevance for immediate application [22]. Stokes’ model adds conceptual insight on the role of relevance when considering the value of research to society, however, it was not intended to specifically conceptualise the term and does not distinguish it from other related concepts such as research impact or value. Therefore, to provide further insights, we next consider relevance in practical settings.

#### Health sector perspectives

In the health sector, the idea that research should be ‘relevant’ is commonplace. Commitments to ‘knowledge translation’ and the ‘knowledge to action cycle’ [23] emphasise issues of relevance and provide considerable insight into approaches to ensuring research usability and use. At the same time, the health research community has given disproportionate attention to issues of research quality, with an emphasis on internal validity that may downplay external validity and suggest some tension between rigour and relevance. Thus, though the concept of relevance is of central importance to the health research enterprise, the failure to unpack it or explore it both theoretically and practically leaves room for misunderstanding and misapplication.

In the health sector, research relevance often arises as a practical question of the ‘fit’ between a body of knowledge or research approach and a specific field or issue (e.g. public health, primary healthcare, healthcare access, genomics, alternative healthcare, healthcare reform in rural areas). The results of two recent International Society for Pharmacoeconomics and Outcomes Research task forces take this approach. The task forces developed questionnaires to assess the relevance and credibility of research other than randomised controlled trials (e.g. observational research, meta-network analysis) to inform healthcare decision-making [24, 25]. Both make similar observations about relevance, reinforcing the subjectivist approach noted earlier, and can be summarised by the following statement by Berger et al. [24]:

“*Relevance addresses whether the results of the study/apply* [sic] *to the setting of interest to the decision maker. It addresses issues of external validity similar to the population, interventions, comparators, outcomes, and setting framework from evidence based medicine.* ***There is no correct answer for relevance. Relevance is determined by each decision maker, and the relevance assessment determined by one decision maker will not necessarily apply to other decision makers.*** *Individual studies may be designed with the perspective of particular decision makers in mind (e.g. payer or provider)*” (p.148, emphasis added).

Research relevance in health is also noted in discussion and debate regarding the value of qualitative research relative to the more established forms of quantitative health research. For example, Mays and Pope [26] suggest that qualitative research can be assessed “…*by two broad criteria: validity and relevance*”. Their further discussion provides some insight into the several ways that research might be relevant, suggesting that:

“[r]*esearch can be relevant when it either adds to knowledge or increases the confidence with which existing knowledge is regarded. Another important dimension of relevance is the extent to which findings can be generalised beyond the setting in which they were generated*” [27].

The work of Mays and Pope positions research relevance amidst the longstanding tension between internal and external validity. This tension reflects opposing foci on internal validity as the quality/rigour of research methodology and external validity as the applicability/transferability of research to other settings or contexts. While external validity is not the only measure of relevance – as research may remain relevant to some contexts even when not generalisable to others – it is an important component, and one that has not always attracted sufficient attention. For example, the Canadian health research community has focused considerable practical attention on internal validity as a critical component of evidence for clinical and health policy decisions. Evidence-based medicine, the Cochrane Collaboration, the Canadian and United States task forces on preventive healthcare/services and a long list of aligned groups have developed and established many tools to assess the quality of research evidence (e.g. GRADE [28]), with a predominant focus on issues of internal validity, and an emphasis on evidence hierarchies that is sometimes seen to be incompatible with ‘real world’ relevance. The relative lack of similar approaches or tools that focus on external validity in health research is notable, though movements to marshal evidence in support of sound public policy, such as the Campbell Collaboration, have attended to issues of external validity in other areas of health and social policy [29]. Further, there are emerging approaches and tools for documenting the external validity of health research and facilitating its use [30]. For example, WHO has supported the development of workbooks to contextualise health systems guidance for different contexts [31] and the field of local applicability and transferability of research has emerged to facilitate the adaptation of interventions from one setting to another, including the development of some well-documented tools like RE-AIM [32].

Alongside these emerging approaches and tools sits the established field of KT. KT has a strong history in Canada with a distinctive feature being a reliance on stakeholder engagement to support a commitment to improve research relevance. For example, the AIHS framework relies heavily on KT and stakeholder engagement approaches as part of its RIA, describing the mobilisation of knowledge through “*…a process of interactions, feedback, and engagement using a variety of mechanisms (e.g. collaborations, partnerships, networks, knowledge brokering) with relevant target audiences (i.e. actors and performers) across the health sector*” ([8] p. 362). Experience in stakeholder engagement, particularly with clinical, management and policy decision-makers, has become fairly extensive and there is now increased attention on engaging patients as core stakeholders in health research. If relevance is truly subjective, then KT efforts (including engagement, dissemination, promotion, communication) would appear to represent reasonable approaches for articulating, conveying and improving research relevance. However, if there are underlying elements of relevance that are more universal, then there is a risk that KT efforts – and subjectivist approaches to ensuring relevance – are akin to commercial marketing or communication strategies where the aim is to ‘sell’ more product and/or generate more influence that may not align with a more objective lens.

In sum, the health research community in Canada has a longstanding history of critically appraising research quality based on study design and research methodology, with greater emphasis on internal rather than external validity. As the same time, there is established expertise in KT, emphasising engagement with research users and adaptation to settings or contexts of use – approaches that may imply a subjectivist interpretation of relevance. Thus, while relevance is an important concept for the health research enterprise, its use is largely tacit and taken for granted.

#### Non-health sector perspectives

To unpack relevance further we consider some non-health sector perspectives that give attention to the term, often with formal definitions or taxonomies established. Examples include the legal, financial accounting, education and web search (information retrieval) sectors, each of which are briefly described below.

From a legal perspective, relevance has a specific meaning that relates to the admissibility of evidence in terms of its probative value (i.e. the extent to which evidence contributes to proving an important matter of fact) [33]. For example, a common objection to legal testimony or evidence is that it is ‘irrelevant’ [34]. Legal processes for considering the admissibility or legal-relevance of evidence are firmly established, requiring explicit declaration of evidentiary sources and direct consideration of that evidence as it relates both to a specific case and related historical precedents, something that is undeveloped in the health sector [35]. It is the formality, explicitness and retrospective nature of this process, which is directly associated with a specific case (or decision), that is characteristic of the consideration of relevance in the legal context.

Financial accounting provides another perspective on relevance. In this field, relevance is viewed as a fundamental component of generally accepted accounting principles. Relevance and materiality are emphasised such that accountants and auditors focus on financial information that meets the decision-making needs of users and is expected to affect their decisions. In financial accounting, ‘value relevance’ provides a more focused perspective on relevance, defined as “*…the ability of information disclosed by financial statements to capture and summarise firm value. Value relevance can be measured through the statistical relations between information presented by financial statements and stock market values or returns*” [36]. Similar to the legal perspective, the financial accounting perspective on relevance is set with a formal context, where the focal point (i.e. financial performance) is clear and principles (i.e. generally accepted accounting principles) and processes (i.e. financial reporting and auditing) are clearly established and monitored.

Education provides a slightly more expansive approach to operationalising relevance, given the more general aim of the enterprise. In the United States, the Glossary of Education Reform [37] notes that “*…the term relevance typically refers to learning experiences that are either directly applicable to the personal aspirations, interests, or cultural experiences of students (personal relevance) or that are connected in some way to real-world issues, problems, and contexts (life relevance)*”. They further state that “*personal relevance occurs when learning is connected to an individual student’s interests, aspirations, and life experiences*”, while “*life relevance occurs when learning is connected in some way to real-world issues, problems, and contexts outside of school*”. A similar framing of relevance in this context suggests that it *“…extends the learning beyond the classroom by teaching students to apply what they are learning to real world situations*” [38]. While the education sector also makes numerous references to a ‘rigour and relevance’ dyad [39] in contrast to the dominance of the internal validity focus in healthcare, it is the prominent dual focus on ‘personal’ relevance (with its subjectivist orientation) and ‘life’ or ‘real world’ relevance (with its more universal orientation) that seems to most clearly define the education sector’s perspective on relevance.

One of the most intensive and competitive sectors focusing on relevance is the web search (or information retrieval) field. This includes dominant search engines such as Google and Bing, as well as a wide range of commercial and social media sites such as Amazon, eBay, Facebook and LinkedIn, that compete either directly or indirectly on their ability to identify relevant information in response to user queries. Therefore, the ability of these organisations to advance the theory and practice related to relevance is fundamental to their success. For example, Google was built upon the effectiveness of its search algorithm, which is in a constant state of evolution. Both explicit and implicit approaches to assess relevance are used to contribute to search algorithm refinements [40]. The explicit approach focuses on ‘relevance ratings’, whereby evaluators (e.g. human raters) are contracted to assess the degree of ‘helpfulness’ of search results paired to specific search queries [41]. The implicit approach to assess relevance monitors and aggregates search behaviour of millions of users who are likely unaware that their behaviour is being assessed. Google has more recently advanced ‘personalised relevance’, which uses past individual search behaviour to personalise/tailor future search results for the same individual. Pariser has critiqued this concept as “*the filter bubble*” [42], warning that Google’s intent to optimise search algorithms for personal relevance creates a “*…personal ecosystem of information…*” that limits the diversity of search results and promotes insularity. This personal relevance is situated within the pervasiveness of social media, which facilitates the advancement of ‘social relevance’. Personal and social relevance highlight two important orientations towards relevance – one built on increasingly detailed understanding of individual preferences and the other reflecting the growing power and increasing accessibility of crowd-sourced perspectives. Overall, web search has made important contributions to how we understand and operationalise relevance, including the use of increasingly sophisticated explicit and implicit feedback mechanisms and the ability to draw upon and analyse big data sets. Web search has also exposed the contrasting orientations of personal and social relevance that underscore the challenges of combining or integrating different relevance assessments.

These non-health sector perspectives on relevance highlight several considerations. First, they reinforce general findings that point to perspective, decision context, timeliness and precision of focus or ‘fit’ as key elements of relevance. Additionally, they highlight a few distinctive considerations. The formalistic contexts of financial accounting and law emphasise issues such as precedent and legitimacy, implying that relevance in a research sense might require the demonstration of some legitimate or credible association between research and its use or user, among other considerations. Further, the complex consumerist world of social media highlights some of the challenges of a purely subjectivist definition of relevance. Whereas the International Society for Pharmacoeconomics and Outcomes Research guidance takes a subjectivist stance in suggesting that, “[t]*here is no correct answer for relevance*” [24], the “*filter bubble*” criticised by Pariser [42] suggests otherwise. Relevance solely to the personally-perceived interests of a research user is unlikely to adequately serve the collective commitments to health and health equity that are especially germane to the health research enterprise.

### Forward-looking considerations for assessing the relevance of health research

To this point, we have endeavoured to unpack relevance from theoretical and practical perspectives. In light of these insights and in the context of persistent interest in research impact assessment and evolving interest in research relevance, we now turn to some specific forward-looking considerations for research relevance assessment (RRA).

#### Relevance of research versus everything else

The first consideration for RRA is the acknowledgement that research is only one of many sources of insight to inform the needs or actions of research users. A research user is influenced by a wide range of political, legal, media, economic and other contextual information, interactions and experiences, as well as prevailing organisational governance, leadership, culture and values that all serve to complement (and often dominate) any insights that might be derived from research [43]. This reality implies that ‘relevance’ has a different meaning for researchers and research users. Researchers are typically interested in the relevance of a specific research product or activity for identifiable actions of (potentially) multiple research users; relevance is here judged relative to both the perceived needs of research users, and the extent and content of other related research. In contrast, research users are typically focused on identifying multiple relevant inputs to guide a specific action, only some of which may be research; relevance is here judged relative to both the research user’s needs and the form and content of the other inputs.

Given these distinct orientations to research relevance, RRA needs to be explicit about its comparative lens. Clear distinctions should be made between relevance based on the merits of the research product or activity (researcher lens) and relevance based on the relative value of research compared to other research and non-research sources (research user lens). RRA provides an opportunity to build more robust ways to characterise and assess the contribution of research to research users, including a more systematic and transparent articulation of anticipated research uses (akin to the Research Councils UK’s ‘Pathways to Impact’ [44] or descriptions of planned study design and methodological approach published in study protocols/registrations for randomised controlled trials or systematic reviews).

#### Beyond instrumental uses of research

The considerations noted above rely heavily on instrumental uses of research. Theoretically derived definitions of relevance, such as Floridi’s [14], tend to focus on the response to a specified question. This suggests a direct and tangible connection between research and its ‘use’. However, as Weiss [43] and others have observed, most types of research use are not instrumental, where use is documented and explicitly addresses a specific query or challenge for a research user. Rather, research use tends to be more conceptual, where use is indirect and evolves over time, or symbolic, where use may be politically or tactically motivated [43]. Research may also create externalities or unintended effects. For example, general research activity might support an engaged learning environment, interactive research relationships, and additional research-related discourse that provides benefits that are not attributable to any specific research product or activity. This has important ramifications for how research is funded and the role that relevance can play in that assessment. Ultimately, RRA needs to go beyond a singular understanding of research use as instrumental use, to develop better methods for capturing and assessing the relevance of the many non-instrumental uses of research.

#### The temporal factor

Another closely related consideration for RRA is the temporal context. Almost all research is conducted in a temporally defined period. Yet, while the quality of research is typically characterised by its methodology, which is a static feature typically not subject to temporal variation (e.g. the assessed quality of a randomised controlled trial should be consistent over time), relevance of research can be considered at any time (e.g. prior to the initiation of a research study or at different points in time post-completion) and is therefore subject to dynamic perceptions as they pertain to evolving action or decision contexts. Cohen [15] suggests that “*…relevance, like reasoning, has a prospective dimension as well as a retrospective one. It helps prediction as well as explanation*” (p. 182). The important insight is that, in contrast to research quality, the relevance of a specific research product can change over time, making assessment of research relevance more challenging.

This requires RRA to acknowledge the temporal factor and its associated implications for research relevance. At minimum, RRA should specify the temporal context as either pre-research (e.g. proposal/funding stage) or post-research (e.g. after research results have been produced). RRA at the pre-research stage focuses on proposed inputs and hypothetical outputs and outcomes, and may be more likely to overestimate instrumental research use and underestimate non-instrumental use. RRA at the post-research stage focuses mainly on the importance and value of actual outputs and tangible results, and may capture more non-instrumental research use. The pre-research stage is clearly aligned with research funding/investment processes, while the post-research stage can contribute to retrospective return-on-investment calculations and more general research impact assessment. However, employing this simple temporal categorisation should not lead us to lose sight of the dynamic, iterative nature of research relevance and the opportunity to assess it at interim and ongoing stages that captures re-interpretations or re-applications of research findings over time.

#### Moving from a subjective to an intersubjective understanding of relevance

An underlying theme in our review of relevance is subjectivity. Consider the broad scientific paradigms of positivism and interpretivism that are typically respectively aligned with research quality and research relevance. Research quality can be viewed as relating to characteristics or features that are assessed objectively, while research relevance may be seen as subjectively adjudicated. The subjective focus emphasises the variability of different perspectives and contexts and the suggestion that anyone can have a different take on the relevance of a specific research product or activity. For RRA, this reinforces a user-centred orientation to relevance assessment that privileges the judgment of the interrogator and raises the key question regarding who is positioned as the main arbiter of research relevance.

However, while relevance may never be characterised as universal, it could be argued that it is not purely subjective either. Rather, relevance may be more consistent with an intersubjective understanding that emphasises the extent of agreement or shared understanding among individual subjective perspectives representing a way to bridge the personal and the universal. The intersubjective view, while not presenting an objective approach to measuring relevance, does provide a road towards a meaningful and structured assessment of research relevance. It also emphasises the importance of representation in forging the intersubjective judgments that guide the research enterprise.

## Conclusions

This paper has unpacked research relevance from different perspectives and outlined key considerations for its assessment. Alongside research impact assessment, research relevance seems increasingly important in justifying research investments and guiding strategic research planning. Indeed, judgments of ‘relevance’ are becoming a key component of the health research enterprise. However, consideration of relevance has been largely tacit in the health research community, often depending on unexplained interpretations of value, fit and potential for impact. Reviewing the various uses of relevance in health research, the concept is sometimes used as a synonym for research impact or positioned as a reliable predictor of later consequence. In many ways, research relevance seems a necessary condition for impact – a process or component of efforts to make rigorous research usable. However, relevance is not a necessary or sufficient condition to achieve impact. We expect that research that is relevant, and thus accountable to specific and legitimate users, will be impactful, but this may not necessarily be the case where other factors intervene. Additionally, we may expect that research that is impactful will be appropriately accountable – but again, this is not necessarily the case. Ultimately, relevance stands apart from research impact. Like rigour, relevance is a complementary but distinctive dimension of what it is that ensures ‘the good’ in health research.

While ‘relevance’ is ever-present, understanding of the concept in terms of health research is emergent and not well codified. To improve our understanding, this paper outlines four key considerations, including how research relevance assessments (1) orientate to, capture and compare research versus non-research sources, (2) consider both instrumental versus non-instrumental uses of research, (3) accommodate dynamic temporal-shifting perspectives on research, and (4) align with an intersubjective understanding of relevance. We believe careful and explicit consideration of research relevance, guided by transparent principles and processes is vital to gauge the overall value and impact of a wide range of individual and collective research efforts and investments. We hope this paper generates more discussion and debate to facilitate progress.

## Abbreviations

AIHS: Alberta Innovates – Health Solutions
CAHS: Canadian Academy of Health Sciences
CHSRF: Canadian Health Services Research Foundation
CIHR: Canadian Institutes of Health Research
KT: knowledge translation
OSSU: Ontario SPOR SUPPORT Unit
RIA: research impact assessment
RRA: research relevance assessment
SPOR: Strategy for Patient-Oriented Research
